# The Emerging Role of Long Noncoding RNAs in Sorafenib Resistance Within Hepatocellular Carcinoma

**DOI:** 10.3390/cancers16233904

**Published:** 2024-11-21

**Authors:** Puneet Vij, Mohammad Shabir Hussain, Sanjaya K. Satapathy, Everardo Cobos, Manish K. Tripathi

**Affiliations:** 1 Department of Pharmaceutical Sciences, St. John’s University, Queens, NY 11439, USA; puneet.vij08@outlook.com; 2 Medicine and Oncology ISU, School of Medicine, The University of Texas Rio Grande Valley, McAllen, TX 78504, USA; mohammad.hussain@utrgv.edu (M.S.H.); everardo.cobos@utrgv.edu (E.C.); 3 South Texas Center of Excellence in Cancer Research, School of Medicine, The University of Texas Rio Grande Valley, McAllen, TX 78504, USA; 4 Department of Medicine, Donald and Barbara Zucker School of Medicine at Hofstra, Northwell Health Center for Liver Diseases & Transplantation, Northshore University Hospital, Manhasset, NY 11030, USA; ssatapat@northwell.edu

**Keywords:** long noncoding RNA (LncRNA), Sorafenib resistance, hepatocellular carcinoma (HCC), EGFR, autophagy, VEGFA, proteomics, renal cell carcinoma (RCC)

## Abstract

Hepatocellular carcinoma (HCC), a liver cancer originating from hepatocytes, is a major health concern and among the most common malignancies worldwide. Sorafenib, approved by the U.S. F.D.A., is the primary first-line treatment for patients with advanced HCC. While the preferred first-line systemic regimen for HCC is immunotherapy with Atezolizumab plus bevacizumab or Tremelimumab-actl + durvalumab, Sorafenib is still an alternative recommended regimen. While some patients with advanced HCC may benefit from Sorafenib treatment, most eventually develop resistance, leading to poor prognosis. Long noncoding RNAs (lncRNAs) have been found to play a critical role in tumorigenesis and the development of HCC, as well as other cancers. They are also key players in tumor drug resistance, though the mechanisms of lncRNAs in Sorafenib resistance in HCC remain poorly understood. This review summarizes the molecular mechanisms contributing to Sorafenib resistance in HCC with their potential correlation with lncRNAs, including the roles of transporters, receptors, cell death regulation, and other influencing factors.

## Simple Summary

Liver cancer is a pressing global health concern, with hepatocellular carcinoma (HCC) being the most prevalent type. It is a leading cause of liver cancer-related deaths worldwide. According to the American Cancer Society (ACS) in 2024, the U.S. will see an estimated 41,630 new cases of liver cancer, with Texas expected to have the second-highest number of liver cancer deaths, particularly among Hispanics, who experience the highest mortality rates. The South Texas Rio Grande Valley (RGV), where the population is approximately 90% Latino/Hispanic, is a major hotspot for cancers influenced by factors like obesity, diabetes, socioeconomic challenges, oxidative and mental stress, and both alcoholic and non-alcoholic fatty liver disease. This study addresses the challenge of Sorafenib resistance in targeted therapy and explores the role of long noncoding RNAs in HCC to improve treatment outcomes, focusing on underserved communities in the Texas Valley.

## 1. Introduction

Liver cancer is a global health problem estimated to have more than one million cases worldwide in 2024. Hepatocellular carcinoma (HCC) is the most prominent and is among the leading causes of liver cancer-related death worldwide; it accounts for approximately 92% of all existing cases and relates to liver cirrhosis to a certain extent. Liver cirrhosis directly affects the safety of the treatment and plays a significant role in determining the prognosis of HCC [1,2]. Sorafenib was approved in 2007 for the treatment of advanced HCC, a milestone in the history of HCC treatment. Sorafenib is a preferred treatment for advanced hepatocellular carcinoma (HCC) [3]. This drug has demonstrated the benefit of survival over supportive care in advanced HCC. In the Sorafenib Hepatocellular Carcinoma Evaluation Randomized Protocol (SHARP) trial, 5.5 months (median time to progression) and 10.7 months (survival) were observed in sorafenib-treated patients, compared to a placebo. The absolute improvement in survival was 2.8 months [4,5,6]. Various studies have shown that patients treated with sorafenib have an increased median survival time [7] and an increased resistance level, significantly limiting the efficiency of sorafenib therapy [8]. This is the primary factor causing hindrances in its clinical application. Long noncoding RNAs (LncRNAs) are non-protein-coding ribonucleic acids (RNAs) with more than 200 nucleotides. LncRNAs have been found to play critical roles in developing various cancers, including HCC [9,10]. Although the role of lncRNAs in disease onset has received attention, studies on the relationship between lncRNAs and chemotherapy resistance, especially in sorafenib-resistant HCC, are scarce. The role of lncRNAs in the pathogenesis and progression of various cancers, including hepatocellular carcinoma (HCC), is crucial. These lncRNAs regulate vital processes such as proliferation, migration, apoptosis, autophagy, the cell cycle, tumorigenesis, and metastasis in HCC and related cancers such as renal cell carcinoma (RCC) [11]. Additionally, because the kidney is located near the liver and often plays a compensatory role in liver disease, RCC is also significantly impacted. We will include relevant information on RCC, particularly in the context of sorafenib resistance. A small percentage of patients can get an advantage from sorafenib, and the same proportion of patients commonly acquire drug resistance within a small amount of time [12]. Patients administered with sorafenib usually experience side effects such as gastrointestinal, physical, or skin-related issues (e.g., skin reactions, weight loss, and/or diarrhea) [13,14]. When the effects are severe, sorafenib can cause high blood pressure and/or abdominal pain, which can lead to this drug’s discontinuation [6,15,16]. The mechanisms of sorafenib resistance should be well established. The role of epigenetics, transport processes, regulated cell death, and the tumor microenvironment in the sorafenib resistance in HCC and RCC have been established based on recent studies [17,18,19]. HCC is a unique cancer type; this typically arises in the setting of chronic liver disease at a rate dependent upon the complex interplay of the host of the liver disease and the environmental factors that are responsible [16,20]. The other cause of infection with chronic hepatitis B or C virus is currently the dominant risk factor worldwide for chronic liver diseases after being converted into HCC [20,21]. This review summarizes the mechanisms mentioned above and the role of lncRNA in developing resistance.

### 1.1. Sorafenib Resistance in HCC

The efficacy of Sorafenib is often hampered by the development of both primary and secondary resistance, which limits its therapeutic success. Sorafenib resistance can be divided into intrinsic (also known as primary) resistance and extrinsic (also known as secondary) resistance. Primary resistance occurs when cancer cells are inherently non-responsive to sorafenib at the outset of treatment. This can be attributed to the genetic heterogeneity of cancer/tumor cells. The reason behind the lack of effectiveness and sensitivity of sorafenib in the early stages of treatment in HCC and RCC is that liver and kidney cancer cells have resistance factors even before sorafenib treatment. The most common type of renal cancer is RCC, and the genes described as altered are V.H.L., PBRM1, SETD2, KDM5C, PTEN, BAP1, mTOR, TP53, TCEB1 (E.L.O.C.), SMARCA4, and ARID1A, frequently reported in RCCtherapy [18,22].

Additionally, the tumor microenvironment plays a significant role in primary resistance. Cancer-associated fibroblasts (CAFs) and other stromal components can secrete growth factors and cytokines that activate alternative signaling pathways, such as the PI3K/AKT pathway, which can bypass the inhibitory effects of sorafenib [23]. Furthermore, epigenetic modifications, such as DNA methylation and histone modifications, can lead to the silencing of tumor suppressor genes and the activation of oncogenes, contributing to intrinsic resistance. Secondary resistance is defined as when cancer/tumor cells become less sensitive to sorafenib after a period of treatment, which ultimately can result in treatment failure [24,25]. Sorafenib resistance limits its therapeutic effect, so gaining a better understanding is paramount. 

One common mechanism of secondary resistance is the acquisition of new genetic mutations. For example, secondary mutations in the BRAF gene can arise during sorafenib treatment, resulting in continued activation of the RAF/MEK/ERK pathway despite the presence of the drug [26]. Moreover, cancer cells can undergo phenotypic changes, such as epithelial-to-mesenchymal transition (EMT), associated with increased invasiveness and drug resistance. EMT is regulated by various transcription factors, which can be induced by prolonged exposure to sorafenib. Another mechanism involves upregulating drug efflux pumps, such as ATP-binding cassette (ABC) transporters, which actively pump sorafenib out of cancer cells, reducing its intracellular concentration and effectiveness [27]. Additionally, cancer cells can activate compensatory signaling pathways to circumvent the inhibitory effects of sorafenib. For instance, increased JAK/STAT3 pathway activity has been observed in sorafenib-resistant HCC cells, promoting cell survival and proliferation. Primary and secondary resistances significantly hinder the therapeutic effectiveness of sorafenib. Thus, it is essential to understand these resistances. The significant determinants involved in sorafenib resistance are presented in Table 1.

### 1.2. Primary Resistance

Primary resistance refers to the inherent insensitivity of cancer cells to sorafenib from the outset of treatment. This resistance arises from pre-existing mechanisms within the tumor cells that enable them to evade the drug’s effects. It has been widely accepted that the initiation and development of HCC are a consequence of complex genetic and epigenetic alternations [28]. Genetic heterogeneity is responsible for resistance factors present in patients’ tumor tissues or HCC cells before the initiation of drug therapy. BRAF mutations can result in primary resistance by maintaining the activation of the MAPK/ERK pathway, which sorafenib targets [29]. Alternative signaling pathways (EGFR, VEGFA) and cellular adaptions (Sestrin 2) also contribute to the primary sorafenib resistance [1,30,31]. By understanding the alternative signaling pathways responsible for sorafenib resistance, researchers can better develop strategies to overcome primary resistance and enhance the efficacy of sorafenib in cancer treatment. 

#### 1.2.1. Epidermal Growth Factor Receptor (EGFR) Activation

Epidermal growth factor receptors (EGFRs) reside on the surface of epithelial cells and are the expression product of the proto-oncogene c-erbB1. After ligand binding, EGFRs can activate a series of downstream signaling pathways, which can help regulate cell growth and proliferation. EGFR overexpression and abnormal activation can be seen in most HCC patients [32,33]. Erlotinib, an oral tyrosine kinase inhibitor of EGFR, has shown moderate antitumor activity against HCC [34]. In a study by Sueangoen et al. (2020), seven HCC-derived EGFR mutants studied were erlotinib-resistant and EGF-dependent. Erlotinib induced autophagy and apoptosis in cells harboring different EGFRs. The inhibition of EGFR phosphorylation by erlotinib was the decisive factor for the degree of apoptosis and autophagy amongst cells harboring EGFR mutants [35]. In another study by Peng et al., the author used a human primary HCC and two human HCC cell lines, i.e., Hep3B and Huh7, to develop three sorafenib resistance HCC cell lines. The authors found that EGFR was significantly augmented in all three SR HCC cell lines. EGFR’s tyrosine kinase activity inhibition observed with erlotinib and short hairpin RNA (shRNA) recovered the response of three SR HCC cell lines with the help of sorafenib, which suggests the crucial roles of EGFR tyrosine kinase and KLF4 in the induction of sorafenib resistance [36,37,38]. 

Several studies have highlighted the role of lncRNAs and associated targets/proteins that are frequently overexpressed in fibrosis, carcinoma, and cell injury due to renal pathologies and are often linked to tumor stage and metastasis [39,40]. The expression patterns demonstrated that lncRNA targets and associated proteins might serve as biomarkers or therapeutic targets for HCC and RCC [39,40]. In addition, the EGFR mutations and the single nucleotide polymorphism (SNP) of Aurora kinase A are associated with earlier tumor stages of several cancers [40,41]. The EGFR, long noncoding RNA H19, LAMC1, and SNP rs3768617 could also increase the risk of cancer, causing the progression, metastasis, and expression with inflammation and oxidative stress markers of lung, liver, renal cancer, and chronic kidney diseases. Specific EGFR mutations, such as L858R and exon 19 in-frame deletions, are correlated with higher sensitivity in HCC, RCC, and lung cancer [39,40]. EGFR inhibition, combined with sorafenib, can effectively suppress HCC progression [42]. Specifically, they found that dual targeting of EGFR and VEGFR2, using the combination of erlotinib and sorafenib, significantly enhanced antitumor effects compared to either drug alone. The study underscores the role of EGFR in driving resistance through crosstalk with the VEGFR2 signaling pathway, suggesting that multi-targeted strategies could yield improved therapeutic outcomes in HCC [43]. Additionally, the potential of a novel EGFR inhibitor, afatinib, in sorafenib-resistant HCC models has been explored. The researchers demonstrated that afatinib suppressed EGFR phosphorylation and modulated the downstream PI3K/AKT and MAPK pathways, both of which contribute to resistance. Their findings suggest that afatinib could be an effective treatment option for overcoming sorafenib resistance by attenuating these critical survival pathways in HCC. LncRNA EGFR-AS1 was found to be upregulated in sorafenib-resistant HCC cells, promoting EGFR pathway activation. Silencing EGFR-AS1 in combination with EGFR inhibitors restored sorafenib sensitivity by downregulating EGFR expression and its downstream signaling components, including AKT and ERK. This suggests that lncRNAs, like EGFR-AS1, could serve as novel therapeutic targets for disrupting EGFR-driven resistance mechanisms. These recent studies reinforce the importance of targeting EGFR and its associated pathways to combat sorafenib resistance in HCC, particularly through combination therapies that address the complex interplay between EGFR and other survival mechanisms [44].

#### 1.2.2. Sestrin 2

Sestrin 2 is a tumor biomarker that plays a key role in tumor development. Some studies have identified sestrin 2 as a tumor suppressor gene, while others have identified it as an oncogene [45]. Sestrin 2 is an essential part of the sestrin stress-induced protein family and participates in the development of tumors [46,47]. It regulates multiple downstream pathways, among which MAPK and AKT are closely related to cell proliferation and metabolism. The upregulation of sestrin 2 demonstrates primary resistance to sorafenib in HCC [31]. This resistance, which limits the efficacy of sorafenib, is a significant barrier to successful treatment outcomes in HCC patients. One of the primary mechanisms by which sestrin 2 confers resistance is through the activation of the PI3K/AKT signaling pathway. Sestrin 2 enhances PI3K/AKT signaling in HCC cells, promoting cell survival, proliferation, and metabolism. Upon activation, PI3K facilitates the phosphorylation of AKT, a central kinase that inhibits pro-apoptotic pathways while supporting cellular growth and energy metabolism. The phosphorylation cascade driven by AKTactivation leads to downstream effects such as increased glucose uptake, enhanced lipid biosynthesis, and inhibition of apoptosis, all of which contribute to the resistance of HCC cells to sorafenib [48,49]. Sestrin 2 mediates this resistance by activating the AKT and AMPK signaling pathways, which are critical for cell survival and metabolism. The upregulation of sestrin 2 in HCC cells enhances the phosphorylation and activation of AKT. This key kinase promotes cell survival by inhibiting apoptosis and supporting metabolic processes essential for cell growth. Sestrin 2 activates AMPK, an energy sensor that helps maintain cellular energy balance under stress conditions induced by sorafenib. This dual activation of AKT and AMPK by sestrin 2 provides a survival advantage to HCC cells, enabling them to resist the cytotoxic effects of sorafenib [31,50]. AMPK activation also inhibits the mTORC1 signaling pathway, which drives protein synthesis and cell proliferation. By inhibiting mTORC1, sestrin 2 creates a feedback mechanism that enables HCC cells to adapt their metabolic demands to environmental stressors, including sorafenib-induced stress [51]. In addition, the knockdown of sestrin 2 increases sensitivity to sorafenib, resulting in higher apoptosis rates and reduced proliferation, underscoring the importance of these pathways in sestrin 2-mediated resistance. Sestrin 2 belongs to the family of stress-response or stress-induced proteins; it has been reported to stress in several organs. Sestrin 2 is associated with decreased glomerular parietal epithelial cells [52], decreased/regulated autophagy, and the promotion of human serum albumin (HSA)-induced epithelial-to-mesenchymal transition (EMT) and endoplasmic reticulum (ER) stress in HK-2 cells [52,53]. Sestrin 2 has also been reported to alleviate fibrosis and ER stress in liver-related diseases [54]. The function of sestrin2 has been studied in high glucose-stimulated mesangial cells, where it is downregulated and induces fibrosis through the AMPK pathway [54]. Additionally, sestrin 2 alleviates liver ER stress via the pathway, contributing to increased ER stress and regulating protein synthesis through eEF2 inactivation [55]. LncRNA CDKN2B antisense RNA1 has been shown to be associated with sestrin 2 in a different study [56].

#### 1.2.3. Vascular Endothelial Growth Factor A (VEGFA)

VEGF is a critical cellular target of sorafenib. It was initially isolated from tumor cells and is involved in glioma cell proliferation, angiogenesis, and metastasis [57]. VEGFA exerts its effects primarily by activating the AKT and AMPK signaling pathways, which play crucial roles in cell survival, proliferation, and metabolism. VEGF stimulates paracrine secretion of hepatocyte growth factor by stromal cells, which promotes tumor progression [1,58]. It is a potent pro-angiogenic factor that promotes the formation of new blood vessels, a process crucial for tumor growth and metastasis. In HCC, VEGFA is often overexpressed, leading to enhanced angiogenesis and tumor progression. VEGFA binds to its receptor VEGFR2, initiating a series of downstream signaling events that activate various cellular pathways involved in proliferation, survival, and resistance to apoptosis. Upon VEGFA binding to VEGFR2, the receptor undergoes autophosphorylation, which activates phosphoinositide 3-kinase (PI3K). Activated PI3K then converts PIP2 to PIP3, which recruits AKT to the plasma membrane, which is phosphorylated and activated. Activated AKT promotes cell survival by inhibiting pro-apoptotic factors such as BAD and by upregulating cell proliferation pathways, enhancing the survival capacity of tumor cells under sorafenib-induced stress [51]. AMPK activation enhances glucose uptake and fatty acid oxidation, providing the energy necessary for cell survival under therapeutic stress. This metabolic adaptation is crucial for the primary resistance of HCC cells to sorafenib. The association between lncRNA and VEGFA has also been studied. lncRNA (UBE2CP3) enhances VEGFAsecretion and promotes angiogenesis in HCC cells by activating ERK1/2/HIF-1α/VEGFAsignaling in hepatocellular carcinoma [59,60]. C-Jun N-terminal kinase; JNK (Mitogen-Activated Protein Kinase; MAPKfamily) JNK (c-Jun N-terminal kinase) is a member of the MAPK (mitogen-activated protein kinase) family that regulates a range of biological processes implicated in tumorigenesis and neurodegenerative disorders. It is also known as the AP-1 transcription factor subunit. It is located at 1p32-p31; the deletion and translocation of this chromosomal have been associated with the development of malignant tumors. In a study performed by Haga et al., the authors demonstrated that the overexpression of c-Jun contributes to sorafenib resistance in HCC [61,62] and the modulation and phosphorylation of c-Jun could be a new therapeutic option for enhancing responsiveness to sorafenib. Preliminary clinical evidence by Chen et al. showed that the prediction of c-Jun activation demonstrates a poor response to sorafenib in HCC [60]. This in vitro study showed that sorafenib treatment could activate the expression of c-Jun, while its inhibition significantly enhanced sorafenib-induced apoptosis in HCC cells. In another study by Hagiwara et al., activation of JNK and a high expression level of CD133 predicted a poor response to sorafenib in HCC. JNK activity was significantly correlated with CD133 expression level, and the high expression level of CD133 was linked to a poor reaction to Sorafenib [63].

LncRNAs contribute to various biological processes by associating with RNA-binding proteins (RBPs) [57]. Understanding the multiple functions of VEGFA and its regulation by lncRNA INC0116 has been shown in glioma tumorigenesis. The novel lncRNA INC01116, when knocked down, suppresses growth, invasion, metastasis, tumorigenesis, and angiogenesis in glioma both in vitro and in vivo by regulating VEGFA expression [57]. It will be interesting to study the role of lncRNA INC01116 in sorafenib resistance, which has not been reported yet.

### 1.3. Secondary Resistance

Secondary resistance results from the resistance factors developed during sorafenib treatment. The tumor cells adapt to continuous drug exposure by upregulating survival pathways and through angiogenesis induction and stress response adaptations. Determinants for secondary resistance are discussed. Elucidating responsible mechanisms may help establish plans to avoid or overcome resistance once it occurs.

#### 1.3.1. Autophagy

Autophagy is a self-degrading system that controls cells to eliminate abnormal proteins and dysfunctional organelles. It plays a crucial role in maintaining homeostasis in cells under stress, such as nutritional deficiency or hypoxia [64]. Based on recent studies, autophagy is a double-edged sword in various cancers by suppressing tumor initiation or supporting their progression [65,66]. This dual outcome mechanism further plays a crucial role in drug resistance, which enables tumor cells to maintain cellular viability under metabolic and therapeutic stress. A study by Feng et al. showed that microRNA (miR-25) increases sorafenib resistance in HCC by inducing autophagy. In addition, this specific microRNA decreases the FBXW7 protein expression to regulate autophagy, which makes miR-25 a peculiar therapeutic target for HCC treatment [66,67]. In another study by Lu et al., the relationship between sorafenib resistance, CD24, and autophagy is described. CD24-associated sorafenib resistance is accompanied by autophagy activation, which can be blocked by inhibiting autophagy using pharmacological inhibitors or knocking down essential autophagy genes. CD24 is a glycoprotein predominantly expressed on the surface of B lymphocytes and other tumors. According to various authors, it is highly expressed in HCC tumor tissues compared with adjacent normal tissues. Furthermore, CD24 expression was significantly increased in patients with residual chemotherapy resistance after sorafenib treatment. Compared to the untreated patients, it suggested that CD24 participates in a sorafenib-induced resistance process, proving that CD24 overexpression in patients was accompanied by autophagy activation [68,69]. lncRNAs are involved in physiological and pathological processes such as development, differentiation, apoptosis, autophagy, inflammation, and cancer. LncRNA HULC triggers autophagy via stabilizing Sirt1 and attenuates the chemosensitivity of HCC cells [70]. LncRNA MALAT1 targets the FOXA1/CD24/Src pathway in human hepatocellular carcinoma [71]. Meanwhile, lncRNA IL21-AS1 facilitates CD24-induced phagocytosis inhibition in ovarian cancer [56]. These might be potential autophagy/CD24 associated lncRNA in a novel mechanism towards sorafenib resistance.

#### 1.3.2. Exosomes

Exosomes are intercellular information carriers and help in regulating the tumor microenvironment. They play a key role in drug resistance by RNA molecules and protein transportation. In HCC and RCC, lncRNAs contribute to various aspects of cancer progression, including tumor initiation, progression, metastasis, recurrence, and drug resistance [72]. They are also packaged and sorted into exosomes, acting as messengers in intercellular crosstalk. Several lncRNAs have recently been shown to regulate exosome biogenesis and secretion in HCC and RCC [72,73,74]. For example, lncRNAs such as HEAIH and HOTAIR facilitate exosome secretion by enhancing the transport of multivesicular bodies toward the plasma membrane in HCC and other cancers. The lncRNA LINC00511 promotes an invasive phenotype in HCC and RCC by increasing exosome secretion. While some lncRNAs have been reported in HCC and RCC, many remain unknown. Exosome-derived lncRNAs may serve as diagnostic and prognostic biomarkers in HCC, RCC, and various other cancers [72,74].

The HCC cell-derived exosomes exerted their functions by increasing the level of proteins related to sorafenib resistance, protecting tumor cells from sorafenib-induced apoptosis, activating the HGF/c Met/Akt pathway in vitro, and targeting the HGF/c-Met/Akt pathway, which may help improve treatment efficacy in liver cancer [75,76]. A study by Liang and Wang demonstrated that sorafenib could promote HCC release of exosomes by enhancing Rab27a activity. Thereby, the secreted exosome promoted the behavior of recipient hepatoma cells and activated the AKT signaling pathway, resulting in decreased sensitivity for chemotherapy [37,77].

#### 1.3.3. Ferroptosis

Ferroptosis was discovered recently and is a novel type of cell death. Increased iron (Fe) accumulation and lipid peroxidation during the cell death process are usually present during the ferroptosis process and are iron-dependent. It is a type of regulated necrosis, distinct from apoptosis (which plays a crucial role in the body’s normal process of maintaining cellular homeostasis [78]), or necroptosis, and is independent of caspase activity and receptor-interacting protein 1 (RIPK1) kinase activity. Tumor cells that evade other forms of cell death are thought to maintain or acquire sensitivity to ferroptosis. Therefore, the therapeutic development of ferroptosis in cancer has received increasing attention [79,80]. More and more studies have shown that the relationship between ferroptosis and cancer is very complex, and ferroptosis is expected to become a new cancer treatment method. During ferroptosis, there are distinct changes in mitochondrial morphology [81], including loss of structural integrity, such as smaller-than-normal mitochondria, condensed mitochondrial membrane density, and reduced mitochondrial cristae [82]. 

The Ras-mitogen-activated protein kinase (MEK) signaling activation can contribute to cancer cells’ sensitivity to ferroptosis. This sensitivity can result from its promotion of iron abundance in cancer by controlling both transferrin receptors and ferritin [81]. Also, microRNA and lncRNAs are increasingly recognized as crucial mediators in ferroptosis regulation. The susceptibility of various types of cancers to ferroptosis is significantly different. The p62-Keap1-Nrf2 pathway plays a key role in rescuing HCC cells from ferroptosis. In addition, the Ras/Raf/MEK pathway is indicated to be a critical target for ferroptosis in HCC treatment [83]. A study by [84] demonstrated that the upregulation of MT-1G by the activation of NRF2 contributes to sorafenib resistance in human HCC cells. Moreover, it is not dependent on kinase inhibition, and the upregulation of MT-1G limits sorafenib-induced lipid peroxidation and subsequent ferroptosis. In addition, blocking MT-1G expression enhances the anticancer activity of sorafenib through the induction of ferroptosis in vitro and in vivo [85]. Another study by Wang et al. investigated the underlying GSTZ1 mechanism of sorafenib-induced ferroptosis in HCC. The authors demonstrated that GSTZ1 was significantly reduced in sorafenib-resistant HCC cells. This reduction further enhanced NRF2 pathway activation and augmentation in the glutathione peroxidase 4 (GPX4) level, which, in turn, suppressed sorafenib-induced ferroptosis. In addition, the sorafenib and GPX4 inhibitor (RSL3) combination significantly inhibited GSTZ1-deficient cell viability and promoted ferroptosis, which resulted from increased ectopic iron and lipid peroxides. The combination of sorafenib and RSL3 has a synergistic therapeutic effect on HCC progression in Gstz1-/- mice in vivo. All these results demonstrated that GSTZ1 can enhance sorafenib-induced ferroptosis by inhibiting the NRF2/GPX4 axis in HCC cells. The authors demonstrated that combination therapy of sorafenib and the GPX4 inhibitor RSL3 may be a promising strategy in HCC treatment [77].

Sorafenib resistance in HCC is often mediated by the activation of alternative survival pathways like PI3K/AKT and compensatory mechanisms that inhibit cell death, including the glutathione (GSH) antioxidant system. Ferroptosis can be exploited by targeting these compensatory pathways, specifically the cystine/glutamate antiporter system Xc−, which plays a key role in regulating intracellular GSH levels. Inhibition of the system Xc− leads to decreased GSH levels, increased R.O.S., and lipid peroxidation, sensitizing HCC cells to ferroptosis [86]. Research has shown that combining sorafenib with ferroptosis inducers, such as erastin, can significantly enhance sorafenib’s therapeutic efficacy by overcoming the resistant tumor cells that escape apoptosis [8,87].

LncRNAs regulate ferroptosis cell death in several cancers. In HCC cells, the high level of lncRNA GABPB1 antisense RNA 1 enhances erastin-induced ferroptosis by blocking the translation of GA-binding protein subunit beta-1 (GABPB1) and suppressing peroxiredoxin-5 peroxidase, leading to reduced cellular antioxidant capacity and cell viability [88]. LncRNA PVT1 has been reported to regulate the expression of ferroptosis-related genes and is associated with sorafenib resistance in HCC. By targeting lncRNA PVT1, studies have shown an increase in ferroptosis sensitivity, suggesting that PVT1 silencing could help overcome resistance [87,89]. The combination of ferroptosis and lncRNA has shown promise in prognostic prediction for HCC and RCC. A prognostic model for HCC has been developed based on ferroptosis-associated differentially expressed lncRNAs, which could be used for prognosis prediction and the selection of cancer groups for therapies [88]. Therefore, ferroptosis-related lncRNAs are potential new therapeutic targets to overcome sorafenib resistance.

#### 1.3.4. Cancer Stem Cells (CSCs)

Cancer stem cells (CSCs) are also known as tumor-initiating cells. These are a small subgroup of cells capable of self-renewal and differentiating characteristics. These CSCs exist in various cancers, including HCC [90]. Recently, it has been demonstrated that CSCs are also involved in therapeutic resistance in HCC, and CSC markers can act as predictors in sorafenib response. A study by Li et al. indicated the role of CSCs in sorafenib-resistant HCC through the IL-6/STAT3 signaling pathway. The authors proved that targeting IL-6 in CSCs is an efficacious therapeutic approach to overcoming acquired resistance. A study by Ohashi et al. showed that ABC transporters can transport a range of toxic substrates from cells and thus directly contribute to resistance. Cscs exhibited increased ABC transporter expression [91].

The eradication of cancer stem cells (CSCs) is emerging as a novel solution to improve the survival rates of HCC and RCC patients. Several signaling pathways are widely recognized as critical mediators of HCC and RCC, contributing to CSC stemness and malignant phenotypes. These pathways are hyperactivated in distinct CSCs and are pivotal for their self-renewal [92]. Additionally, the expression of various lncRNAs is associated with CSCs [93].

#### 1.3.5. Hypoxia

The tumor microenvironment plays a vital role in the development of tumors. Anti-angiogenic drugs can cause blood vessel contraction in the tumor and, in turn, reduce the blood flow. Reduction in blood flow results in oxygen deprivation within the tumor. Based on recent studies, hypoxia in tumors is associated with chemotherapy failure, the selection of more invasive and resistant clones, and/or poor prognosis. Hypoxia plays a vital role in the development as well as the progression of tumors. It has also been implicated in developing drug resistance and activating tumor metastasis. Continuous treatment with sorafenib results in the inhibition of the tumor’s anti-angiogenic activity, which can subsequently induce hypoxia in the tumor. This further promotes the selection of resistant cell clones to adapt to hypoxic conditions, thereby limiting the efficacy of sorafenib. 

A study by Liang et al. [94,95] demonstrated that hypoxia induced by continued sorafenib treatment resulted in sorafenib resistance via HIF-1α/NF-κB activation in HCC EF24, a molecule with structural similarities to curcumin, which could synergistically augment sorafenib’s antitumor effects and help overcome sorafenib resistance by HIF-1α inhibition [96]. Another study by Liao et al. showed that HSP90α plays a key role in sorafenib resistance under hypoxia by blocking necroptosis. The author proved that HSP90α binds with the RIPK1/RIPK3/MLKL complex to induce autophagy, which would be the leading cause of sorafenib resistance. 17-allylamino-17-demethoxygeldanamycin (17-AAG), a specific inhibitor of HSP90α, was able to overcome sorafenib resistance in HCC [97]. Zhao et al. demonstrated that sorafenib upregulates HIF-2α by switching the hypoxia response from HIF-1α to HIF-2α-dependent pathways, resulting in the activation of the TGF-α/EGFR pathway, which contributes to the resistance of HCC cells to sorafenib [98]. These studies proved a relationship between high HIF expression and sorafenib resistance, demonstrating that hypoxia impacts sorafenib treatment and suggested hypoxia induction as a promising approach to overcome resistance.

Hypoxia-mediated lncRNAs have been demonstrated to induce tumor metastasis. Recent studies have increasingly focused on the relationship between hypoxia, lncRNAs, and several cancers [99]. For example, hypoxia-induced TUFT1 is proposed to facilitate HCC growth and metastasis by activating the Ca(2+)/PI3K/AKT pathway [99], and hypoxia-induced HMGB1 mediates HCC and RCCtumor growth via the Toll-like receptor [100]. Current data show that hypoxia-induced HMGB1 boosts HCC tumor invasiveness and metastasis by modulating macrophage-derived IL-6. The liver-specific putative lncRNA, AC115619, is also expressed at low levels in HCC. Both AC115619–22aa and AC115619 play crucial roles in tumor progression and serve as prognostic indicators in HCC and RCC [100].

#### 1.3.6. ATP-Binding Cassette (ABC) Transporters

**Figure 1:**
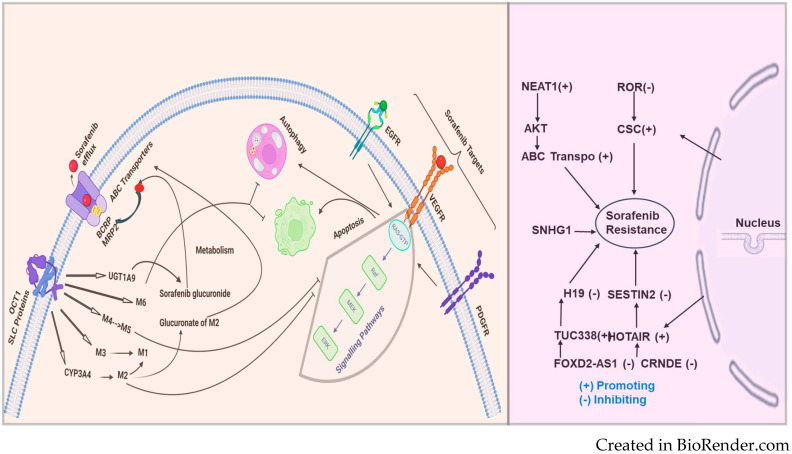
Sorafenib, an anticancer drug, is transported into the cell through SLC proteins (OCT1 and FKSG16) and undergoes metabolism via CYP3A4 (phase 1) and phase 2 U.D.P. glucuronosyltransferase 1A9 (UGT1A9) to form the M1-M8 metabolites. Among the metabolites of sorafenib, M2, M4 (demethylation), and M5 (oxidative metabolite) were found to inhibit vascular endothelial growth factor receptors (VEGFRs). Sorafenib resistance: On the left side of the figure, sorafenib, an anticancer drug, is taken up into the cell primarily through the solute carrier (SLC proteins) and the organic cation transporter (OCT1). Once inside, sorafenib undergoes metabolism via CYP3A4 (phase 1) and phase 2 U.D.P. glucuronosyltransferase 1A9 (UGT1A9) to form the M1-M6 metabolites, including sorafenib glucuronide. These metabolites, particularly M2, can undergo glucuronidation to further reduce their activity. Sorafenib and its metabolites are also effluxed out of the cell by ABC transporters, including BCRP and MRP2, which contribute to drug re-sistance.

ATP-binding cassette (ABC) transporters form one of the most prominent protein families with diverse physiologic functions. These transporters comprise a ubiquitous superfamily of integral membrane proteins responsible for the ATP-powered translocation of many substrates across membranes. ABC-mediated drug efflux is the primary mechanism of multidrug resistance (MDR). The MDR-related transporters in the ABC transporter family include P-glycoprotein (P-gp), breast cancer resistance protein (BCRP), and multidrug resistance-associated proteins (MRPs) [101]. ABC transporter overexpression is a significant cause of MDR [102]. In a study by Zhu et al., the author demonstrated that heme oxygenase 1 (HMOX1) reduces the sensitivity of HCC cells to sorafenib via regulation of the ABC transporter’s expression [103]. A study by Huang et al. investigated the role of BCRP/ABCG2 in HCC sensitivity to sorafenib and showed that BCRP/ABCG2 mediated sorafenib’s efflux and made it resistant. The author also demonstrated that the cotreatment with a BCRP/ABCG2 inhibitor significantly increased the sorafenib cytotoxicity in HCC cells [104]. Figure 1 illustrates the transport of sorafenib and the pathways involved. 

The dysregulated overexpression of ABC transporters mediated by lncRNAs in chemo-resistant cancers is significant and cannot be overlooked. Understanding the underlying mechanisms may provide a theoretical basis for the clinical therapy of HCC and RCCRecent approaches for gene therapy targeting lncRNAs to suppress ABC transporters show promise in reversing HCC and RCC chemoresistance [105]. HULC lncRNA has been reported to correlate with Sirt1 protein levels in human HCC tissues positively and can be used in RCCfor stabilizing Sirt1 protein and triggering autophagy to attenuate the chemosensitivity of HCC and other cancer cells. In oxaliplatin-resistant HCC cells (Huh7/O.X.A. and HepG2/OXA), NR2F1-AS1 knockdown reduces the mRNA expression levels of drug resistance-related genes, including MDR1, MRP5, and LRP1, indicating that NR2F1-AS1 silencing could decrease oxaliplatin resistance. Additionally, the ABCC1 protein is upregulated in oxaliplatin-resistant HCC cells. ABCC1, a known multidrug resistance-related protein, serves as an effective indicator of drug resistance. Both NR2F1-AS1 and ABCC1 are upregulated in these cultured oxaliplatin-resistant HCC cells, suggesting that ABCC1 might function as a direct target of NR2F1-AS1 in regulating oxaliplatin resistance [106,107]. LncRNAs such as MALAT-1, HULC, and H19 have been implicated in human HCC, RCC, and other cancers; however, the functional contributions of these and other lncRNA genes remain largely unknown [108]. MALAT1 was demonstrated to act as a competing endogenous RNA (ceRNA) for miR-216a-5p, thereby preventing the microRNA from inhibiting ABCG2 expression. The upregulation of ABCG2 due to MALAT1 overexpression resulted in decreased intracellular sorafenib concentrations and diminished drug efficacy [43]. This mechanism highlights the complex regulatory network involving lncRNAs and microRNAs that modulate ABC transporter activity and drug resistance. lncRNAs can also epigenetically regulate ABC transporters through histone modification and chromatin remodeling. LncRNA PVT1, for example, has been shown to interact with chromatin-modifying complexes to promote the transcription of ABCC1 (MRP1), a major transporter involved in drug efflux and MDR. In HCC, PVT1 binds to the promoter region of ABCC1 and recruits epigenetic modulators, such as histone methyltransferases, to activate its transcription. This upregulation of ABCC1 contributes to the efflux of sorafenib and other chemotherapeutic agents, resulting in reduced drug sensitivity [42]. By targeting PVT1 or its associated epigenetic machinery, it may be possible to downregulate ABCC1 expression and reverse drug resistance in HCC.

**Figure 2:**
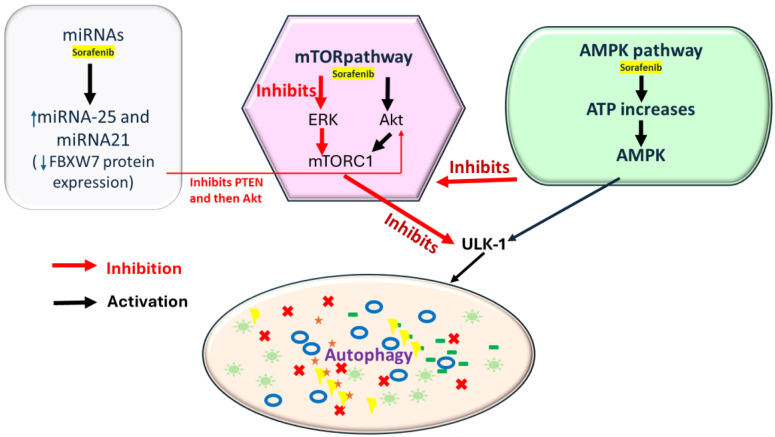
Sorafenib induces the AMPK (AMP-activated protein kinase) pathway by increasing the ATP, initiating autophagy via AMPK, and through mTORC1 inhibition or ULK1 activation. Sorafenib can inhibit mTORC1 via the ERK pathway by inhibiting PTEN (Phosphatase and Tensin Homolog), but it can also activate mTORC1 through Akt activation (key regulators of P13K/AKT/mTOR pathway); it also inhibits ERK, further reducing mTOR activity. In addition, miRNAs are involved in sorafenib-mediated autophagy regulation. Sorafenib increases miR-21 and 25 expression, suppressing autophagy and decreasing FBXW7 protein expression, downregulating PTEN expression, and subsequently activating Akt.

The mechanism of sorafenib resistance and the involvement of lncRNAs are depicted in Figure 2. The figure illustrates the multifaceted mechanisms of sorafenib resistance. OCT1 (SLC proteins) is shown to facilitate the uptake of sorafenib into cells, while ABC transporters (BCRP, MRP2) mediate its efflux, thus regulating intracellular sorafenib levels. The metabolism of sorafenib through enzymes like CYP3A4 and UGT1A9 produces various metabolites, which can affect the efficacy of sorafenib. Key targets of sorafenib, such as EGFR, VEGFR, and PDGFR, are involved in critical cell signaling pathways that control survival and proliferation. The processes of autophagy and apoptosis are shown to influence resistance. The figure also highlights the role of several long noncoding RNAs (lncRNAs)—including NEAT1, ROR, SNHG1, H19, SESTIN2, TUC338, HOTAIR, CRNDE, and FOXD2-AS1—which can either promote or inhibit resistance to sorafenib. Together, these components and pathways illustrate the multifactorial nature of sorafenib resistance, emphasizing the need for a comprehensive understanding of these mechanisms to improve therapeutic strategies.

## 2. Long Noncoding RNAs (lncRNAs) in Sorafenib Resistance

LncRNAs are critical in the sorafenib resistance in HCC. They are longer than 200 nucleotides with no protein-coding ability, and are involved in fundamental biological processes and diverse activities. They can act as a sponge for a variety of miRNAs and also interact with one or more RNA-binding proteins (RBPs) to be involved in multiple biological processes by regulating cell proliferation, apoptosis, metastasis, and invasion. LncRNAs play multifaceted roles in drug resistance across various cancers, influencing cellular processes and contributing to treatment efficacy or resistance mechanisms. Several studies have demonstrated that lncRNAs function as miRNA sponges to regulate target gene expression, mediating sorafenib resistance in HCC. In a study by Niu et al., lncRNA NEAT1 targets miR-149-5p and reduces the activity of sorafenib in HCC cells. NEAT1 was inversely correlated with miR-149-5p expression. Patients with high expression of NEAT1 had worse overall survival rates [24]. In another study by Chen et al., the same lncRNA targeted miR-335 (negatively regulated by NEAT1) and further suppressed the c-Met-Akt pathway, the activation of which leads to drug resistance in HCC cells [109]. Schultheiss et al. demonstrated that more significant amounts of HCC tissue contained reduced levels of this epigenetically regulated lncRNA H19. This lncRNA showed tumor-suppressive actions, and restoring H19 actions might represent an approach for future HCC therapies [110]. Another lncRNA, TUC338, was shown to play a role in the chemotherapy resistance of HCC cells in both in vitro and in vivo studies, which may be mediated by its regulation of RASAL1 expression [111]. A study by Takahashi et al. showed how TGFβ might contribute to altered responses to treatment by assessing the involvement and mechanistic contribution of extracellular vesicle lncRNA in mediating TGFβ-dependent chemoresistance [112]. HOXA13, a HOX gene, is most overexpressed in HCC and is known to be directly regulated by the lncRNA HOTTIP, and high expression of HOXA13 correlates with poorly differentiated hepatocellular carcinomas and increases sorafenib response in vitro models [113]. In a study by Zhang et al., the author showed a correlation of SNHG3 with malignant status and poor prognosis in hepatocellular carcinoma, and SNHG3 expression was directly proportional to tumor size, portal vein tumor thrombus and relapse [114]. Another study demonstrated that the knockdown of the lncRNA SNHG16 attenuated sorafenib resistance in HCC by sponging miR-140-5p, indicating that SNHG16 might be a promising therapeutic target [115]. A study by Sui et al. revealed that the lncRNA FOXD2-AS1 is a vital regulator of TMEM9. FOXD2-AS1 functioned as a sponge for miR-150-5p to modulate TMEM9 expression and contributed to sorafenib resistance [116]. Another study demonstrated that the lncRNA SNHG1 contributed to sorafenib resistance through Akt pathway activation, and its expression is promoted by miR-21, whose nuclear translocation is induced by sorafenib [117]. There are lncRNAs involved in various processes linked to sorafenib resistance, such as regulation of drug efflux and metabolism [118], cellular survival and apoptosis [119], epithelial-mesenchymal transition [120], and epigenetic regulation [121]. There are other studies summarized for HCC in Table 2 whereas Table 3 depicts the studies for RCC. Table 4 includes the studies for both RCC and HCC.

## 3. Conclusions and Future Directions

This study highlights several lncRNAs that play pivotal roles in HCC progression and sorafenib resistance. Notable examples include HULC, MALAT1, H19, NR2F1-AS1, and FOXD2-AS1, each of which contributes to chemoresistance through different molecular mechanisms, such as regulating drug efflux transporters (e.g., ABC transporters) or modulating apoptosis and autophagy pathways. Additionally, lncRNAs like NEAT1, SNHG1, and UBE2CP3 are implicated in controlling angiogenesis and metabolic adaptations, further underscoring their involvement in sorafenib resistance. Sorafenib is the first-line treatment targeted drug for patients with renal cell carcinoma (RCC) as a tyrosine kinase inhibitor. Hence, kidneys are the nearest organ of the liver, and due to severe forms of liver disease or HCC, kidneys are also affected/damaged. Therefore, in this study, we have presented a precise message about sorafenib resistance, which is extremely common in HCC and RCC therapy, and we have shown the molecular mechanisms of sorafenib in HCC and RCC progression. The development of sorafenib resistance in HCC and RCC cells in vitro is essential for exploring the acquired sorafenib resistance mechanisms. Several studies have identified proteins differentially expressed in established sorafenib-resistant HCC and RCC cells compared to their parental cells. 

### Relevance of Sorafenib Resistance in HCC

Sorafenib has long been a mainstay in the treatment of advanced HCC. However, the development of resistance to sorafenib presents a significant hurdle in effectively managing this aggressive cancer. Understanding the mechanisms behind sorafenib resistance is crucial for devising more effective therapeutic strategies and improving patient outcomes. Research into sorafenib resistance in HCC has uncovered several key mechanisms. One major pathway involves the activation of alternative signaling pathways that circumvent sorafenib’s inhibitory effects on the RAF/MEK/ERK pathway. For example, upregulation of the PI3K/AKT/mTOR pathway has been identified as a driver of sorafenib resistance, promoting tumor cell survival and proliferation despite treatment [141,142]. Furthermore, resistance can arise due to dysregulated angiogenesis, a process vital for cancer progression. Increased expression of pro-angiogenic factors such as VEGF and bFGF can lead to enhanced tumor vascularization, thereby reducing the efficacy of sorafenib’s anti-angiogenic effects [143,144]. The tumor microenvironment also plays a critical role in mediating sorafenib resistance. Stromal cells like cancer-associated fibroblasts (CAFs) and tumor-associated macrophages (TAMs) secrete cytokines and growth factors that promote tumor growth and resistance to sorafenib [50,145].

Moreover, genetic mutations and epigenetic modifications contribute to sorafenib resistance by altering drug metabolism, apoptosis, and DNA repair mechanisms. Mutations in genes encoding drug transporters or metabolizing enzymes can affect sorafenib uptake and metabolism, while epigenetic changes can modulate gene expression involved in drug sensitivity. Overcoming sorafenib resistance necessitates the development of innovative therapeutic approaches. Combination therapies, such as targeting multiple signaling pathways simultaneously or combining anti-angiogenic agents with immunotherapy, show promise in preclinical and clinical studies [146]. LncRNA-targeted therapies hold great potential as novel approaches for overcoming sorafenib resistance. Based on current studies, lncRNAs could serve as biomarkers for diagnosis and prognosis and as therapeutic targets to modulate resistance pathways. Specifically, lncRNA silencing or inhibition strategies, such as antisense oligonucleotides or CRISPR-Cas9-based gene editing, show promise in preclinical models. Targeting key lncRNAs involved in regulating PI3K/AKT/mTOR, AMPK, and ABC transporter pathways could enhance sorafenib’s efficacy in resistant HCC.

Future directions in studying sorafenib resistance involve advancing our understanding of the complex mechanisms underlying resistance and developing novel strategies to overcome them. To summarize, the key areas for further investigation include the identification of biomarkers using genomic and proteomic profiling, utilizing sequencing technologies, and liquid biopsies exploring the circulating tumor DNA (ctDNA) and circulating tumor cells (CTCs). Other key areas include mechanistic studies, including single-cell analysis and microenvironment interactions; targeted therapies and combinatorial approaches, including alternate pathway inhibition and immunotherapy combinations; epigenetic regulation, primarily epigenetic modifications in regulating sorafenib sensitivity and resistance; and drug delivery and pharmacokinetic approaches, including nanomedicine approaches and pharmacokinetic studies. Finally, this can be achieved through patient stratification and personalized medicine, including predictive models and clinical trial design. In summary, sorafenib resistance poses a significant challenge in treating advanced HCC. Understanding the diverse mechanisms of driving resistance is essential for devising effective treatment strategies to improve patient outcomes in this lethal disease. Moreover, this review provides strong evidence and systematically summarizes the molecular mechanisms and vital role of the impact of lncRNAs on the sorafenib resistance of HCC and ultimately explores the potential of lncRNAs as new predictive biomarkers and therapeutic targets for HCC. In the future, we shall interpret our ongoing data on sorafenib resistance with integrative studies using biochemical approaches and mass spectrometry (LC-MS) to identify and quantify the novel lncRNA-associated proteome. We have already completed the LC-MS analysis of lncRNA-associated proteins and are finalizing the data for further related manuscripts. We also plan to investigate mouse models for in vivo studies, bioinformatics analysis based on proteomics identification, and pathway analysis. Advancing research in these areas will deepen our understanding of sorafenib resistance mechanisms and pave the way for developing more effective therapeutic strategies. Collaborative efforts across disciplines, the integration of cutting-edge technologies, and patient-centered approaches are essential to overcoming the challenges posed by sorafenib resistance and improving outcomes for cancer patients. LncRNA-targeted therapies represent an exciting frontier in HCC treatment. While further research is needed to unlock their therapeutic potential fully, integrating cutting-edge technologies and patient-centered approaches will be pivotal in overcoming the challenges posed by sorafenib resistance and improving outcomes for HCC patients. Some patients with advanced HCC and RCC may benefit from sorafenib treatment, but eventually develop resistance, leading to poor prognosis. 

LncRNAs have been found to play a critical role in tumorigenesis and the development of HCC RCCas well as several other types of cancers and are key players in tumor drug resistance though the mechanisms of lncRNAs in sorafenib resistance remain to be fully elucidated.

## Figures and Tables

**Table 1:** Major factors contributing to sorafenib resistance at the primary (genetic factors) and secondary (during sorafenib treatment) levels are depicted.

Sorafenib Resistance	
Primary Resistance	Secondary Resistance
EGFR	Autophagy
VEGFA	Exosomes
Sestrin 2	Ferroptosis
JNK (MAPK Family)	Cancer stem cells
	Hypoxia
	ABC Transporters
	LncRNAs

**Table 2:** Studies identifying the lncRNA, including the target and mechanism of action.

Long Noncoding RNAs	Effects on Sorafenib Resistance	Target	Mechanisms Mediating Resistance/Major Effects	Reference
NEAT1	Promoting	miR-149-5p	LncRNA NEAT1 modulates sorafenib resistance in hepatocellular carcinoma by regulating the miR-149-5p/AKT1 axis	[122]
		miR-335	Mediating sorafenib resistance by suppressing miR-335 expression, and dis-inhibition on the c-Met-Akt signaling pathway	[123]
H19	Inhibiting	miR-675	Overexpression of H19 can reduce cell proliferation to reduce chemical resistance after sorafenib treatment	[124]
TUC338	Promoting	RASAL1	TUC338, a lncRNA which is overexpressed in liver cancer and may act as a tumor inducer, to illustrate the function of lncRNA in the development process of chemoresistance in liver cancer in vitro and in vivo. Functionally involved in sorafenib resistance hepatocarcinoma cells by targeting RASAL1	[125]
ROR	Inhibiting	TGF-β	Sorafenib increases expression of ROR in vesicles inside and outside tumor cells, while siRNA to ROR increases sensitivity to chemotherapy	[126]
HOTTIP	Inhibiting	HOXA13	Stable overexpression of HOXA13 in liver cancer cell lines increases cancer cell proliferation and migration and reduces its sensitivity to sorafenib	[127]
SNHG3	Promoting	miR 128	Inducing HCC cells EMT via miR 128/CD151 cascade activation	[87]
SNHG16	Promoting	miR-140-5p	Functioning as an endogenous sponge for miR-140-5p and the effects of SNHG16 knockdown on SR could be blocked by miR-140-5p inhibitor	[122]
FOXD2-AS1	Inhibiting	miR-150-5p	Overexpression of FOXD2-AS1 overcame the resistance of SR cells by functioningas a sponge for miR-150-5p to modulate TMEM9 expression	
SNHG1	Promoting	miR-21	LncRNA SNHG1 contributes to sorafenib resistance by activating the Akt pathway and is positively regulated by miR-21 in hepatocellular carcinoma cells	[43]
HOTAIR	Promoting	miR-217	LncRNA HOTAIR contributes to sorafenib resistance by suppressing miR-217 in hepatic carcinoma	[128]
TRERNA1	Promoting	miR-22-3p	TRERNA1 upregulation mediated by HBx promotes sorafenib resistance and cell proliferation in HCC via targeting N.R.A.S. by sponging miR-22-3p	[129]
TTN-AS1	Promoting	miR-16-5p	LncRNA TTN-AS1 intensifies sorafenib resistance in hepatocellular carcinoma by sponging miR-16-5p and upregulation of cyclin E1	[87]
HEIH	Promoting	miR-98-5p/PI3K/AKT	LncRNA H.E.I.H. confers cell sorafenib resistance in hepatocellular carcinoma by regulating the miR-98-5p/PI3K/AKT pathway	[130]
CRNDE	Inhibiting	miR-543	LncRNA C.R.N.D.E. promotes ATG4B-mediated autophagy and alleviates the sensitivity of sorafenib in hepatocellular carcinoma cells	[87]

**Table 3:** Studies identifying the lncRNAs target, mechanism in RCC.

Long Noncoding RNAs	Effects on Sorafenib Resistance	Target	Mechanisms Mediating Resistance/Major Effects	Reference
LUCAT1	Promoting	miR-495-3p, SATB1, cyclin D1, CDK4, p-Rb, AKT	LUCAT1 significantly inhibits RCC cell proliferation, migration, and invasion, and can lead to the accumulation of renal cancer cell lines	[84]
ARSR	Promoting	miR-34/miR-449 and AXL/c-Met axis	ARSR promotes resistance in RCC and may also be packaged into exosomes to transfer drug resistance	[42]
DMDRMR	Promoting	miR-378a-5p/DAB2IP axis	Promotes angiogenesis	[42,131]
GAS5	Inhibiting	miR-21/SOX5 axis	Inhibit sorafenib resistance	
PLK1S1	Promoting	miR-653/CXCR5 axis	Promotes proliferative and invasive features, as well as sorafenib resistance in RCCcells by regulating C-X-C motif chemokine receptors 5 (CXCR5) by acting as a sponge for miR-653	
SARCC	Inhibiting	AR	Targeted therapy inhibiting invasion, migration, and proliferation in RCC	[132]
SRLR	Promoting	NF-κB/IL-6/STAT3 axis	SRLR directly interacts with nuclear transcription factor NF-κB and binds to and enhances IL-6 transcription, activating STAT3 and promoting sorafenib resistance	[133]
SNHG12	Promoting	Sp1/CDCA3 axis	SNHG12 has been described as a critical molecule mediating tumor development in various tumors, including RCC	[42,134]
KIF9-AS1	KIF9-AS1	KIF9-AS1	KIF9-AS1 sponges miR-497–5p to activate TGF-β and autophagy signaling pathways, promoting RCC cell resistance to sorafenib	[42,135]

**Table 4:** Mechanisms of Primary and Secondary Resistance in HCC and RCC.

Cancer Type	Resistance Type	Mechanism	References
HCC	Primary	Altered drug metabolism (CYP450 enzymes)	[136]
HCC	Primary	Upregulation of drug efflux pumps (MDR1, ABCB1)	[122]
HCC	Primary	Activation of AKT/mTOR pathway	[137]
HCC	Secondary	Epithelial to mesenchymal transition (EMT)	[44]
RCC	Primary	Inactivation of pro-apoptotic signals (p53 mutations)	[138]
RCC	Primary	Activation of alternate survival pathways (VEGF, mTOR)	[87]
RCC	Secondary	Tumor microenvironment modifications (hypoxia)	[139]
RCC	Secondary	Enhanced DNA repair mechanisms (BRCA1, BRCA2)	[140]
